# Arginine Inhibits Adsorption of Proteins on Polystyrene Surface

**DOI:** 10.1371/journal.pone.0070762

**Published:** 2013-08-13

**Authors:** Yui Shikiya, Shunsuke Tomita, Tsutomu Arakawa, Kentaro Shiraki

**Affiliations:** 1 Faculty of Pure and Applied Sciences, University of Tsukuba, Tsukuba, Ibaraki, Japan; 2 Graduate School of Arts and Sciences, The University of Tokyo, Meguro, Tokyo, Japan; 3 Alliance Protein Laboratories, San Diego, California, United States of America; Universidad de Granada, Spain

## Abstract

Nonspecific adsorption of protein on solid surfaces causes a reduction of concentration as well as enzyme inactivation during purification and storage. However, there are no versatile inhibitors of the adsorption between proteins and solid surfaces at low concentrations. Therefore, we examined additives for the prevention of protein adsorption on polystyrene particles (PS particles) as a commonly-used material for vessels such as disposable test tubes and microtubes. A protein solution was mixed with PS particles, and then adsorption of protein was monitored by the concentration and activity of protein in the supernatant after centrifugation. Five different proteins bound to PS particles through electrostatic, hydrophobic, and aromatic interactions, causing a decrease in protein concentration and loss of enzyme activity in the supernatant. Among the additives, including arginine hydrochloride (Arg), lysine hydrochloride, guanidine hydrochloride, NaCl, glycine, and glucose, Arg was most effective in preventing the binding of proteins to PS particles as well as activity loss. Moreover, even after the mixing of protein and PS particles, the addition of Arg caused desorption of the bound protein from PS particles. This study demonstrated a new function of Arg, which expands the potential for application of Arg to proteins.

## Introduction

Nonspecific adsorption of proteins on solid surfaces such as vessels including disposable test tubes and microtubes frequently results in protein denaturation, leading to their inactivation as well as loss in solution. Such protein adsorption occurs during purification, shipping, storage, and routine experiments, and thus presents a serious problem in biotechnology fields, where protein recovery, stability, and purity are of fundamental importance. For example, it has been reported that immunoglobulin G is adsorbed on Teflon [1] and stainless steel [2], leading to disruption of its tertiary structure and irreversible aggregation. Adsorption of lysozyme and ribonuclease A (RNase A) on silica resulted in loss of enzyme activity [3], [4]. Detergents have been commonly used to suppress such surface adsorption, but have disadvantages: they bind strongly to proteins and are difficult to remove from protein solutions due to micelle formation. In addition, various solution additives have been reported to suppress surface adsorption [5]–[8]. For example, more than 3.0 M glycerol and urea were required to reduce the adsorption of RNase A by half at silica-water [5] and air-water [6] interfaces. Salts [7] and sugars [8] also decreased the amount of adsorbed RNase A on polystyrene surfaces at molar concentrations. It should be noted that these additives are effective only at high concentrations. To develop more effective additives that diminish protein adsorption to solid surfaces, especially vessels such as disposable test tubes and microtubes, we tested arginine (Arg), which is now widely used as an aggregation suppressor of proteins [9]–[13].

Arg was first used as a solution additive to increase refolding yield of recombinant proteins, including human tissue type plasminogen activator [14], immunoglobulin [15],[16], interleukin-6 [17], and interleukin-21 [18]. We have also shown that Arg enhances refolding of monomeric [10] and oligomeric proteins [19], and inhibits heat-induced aggregation [9],[10],[12],[13]. It has been suggested that such effects of Arg are due to the ability to solubilize aggregation-prone denatured proteins [20], as also indicated by the enhancement of solubility of hydrophobic small compounds by Arg [21]–[24]. Due to this function, Arg facilitates protein crystallization [11], purification [14], [25]–[27], and formulation [20],[28]. In addition to the prevention of protein-protein interactions, Arg also suppresses binding between proteins and column resins, improving the performance of chromatography [29]–[37]. For example, Arg suppresses nonspecific binding of monoclonal antibodies to the column resins in Protein-A column [29], gel permeation [30], affinity column [31], ion-exchange [32], and MEP HyperCel [37] chromatography. In the present study, to expand its application, we investigated the effects of Arg on protein adsorption to particles 2 μm in diameter made of polystyrene, which is a commonly-used material for vessels such as disposable test tubes and microtubes. Proteins used in this study were albumin, α-chymotrypsin, lysozyme, RNase A, and subtilisin, which differ in size, isoelectric point, and tertiary structure. We examined the effects of amino acids, inorganic salts, and sugar on the adsorption of proteins on polystyrene particles. The results indicated that Arg not only prevents the adsorption of proteins but also desorbs proteins bound to the polystyrene surface.

## Materials and Methods

### Materials

Hen egg white lysozyme, bovine pancreatic ribonuclease A (RNase A), bovine pancreatic α-chymotrypsin (ChT), bovine serum albumin (BSA), *Bacillus licheniformis* subtilisin, polystyrene particles (PS particles) with a diameter of 2 μm, and *N*-succinyl-l-phenyl-alanine-*p*-nitroanilide (SPNA) were from Sigma Chemical (St. Louis, MO). NaH_2_PO_4_ was from Nacalai Tesque (Kyoto, Japan). *Micrococcus luteus*, l-arginine hydrochloride (Arg), guanidine hydrochloride (Gdn), l-lysine monohydrochloride (Lys), NaCl, glycine (Gly), d-glucose (Glc), and dimethyl sulfoxide (DMSO) were from Wako Pure Chemical Industries (Osaka, Japan). Ethanol was from Kanto Chemical Co., Inc. (Tokyo, Japan). All the chemicals used were of high-quality analytical grade.

### Enzyme assay

Enzyme activity was determined as follows. (i) Lysozyme: A substrate solution of 1980 μL containing 0.4 mg/mL *M. luteus* in 10 mM Na-phosphate buffer (pH 7.0) was mixed with 20 μL of protein solution. The enzyme activity was estimated from the slope of the initial decrease in the absorbance at 600 nm. (ii) ChT: A substrate solution of 30 μL containing 20 mM SPNA in a 1∶9 solution of DMSO: ethanol was mixed with 195 μL of 10 mM Na-phosphate buffer (pH 7.0) and 75 μL of protein solution. The enzyme activity was estimated from the slope of the initial increase in the absorbance at 410 nm.

### Adsorption of protein on polystyrene particles

A protein solution of 20 μL was first mixed with 220 μL (for lysozyme, ChT, BSA, and subtilisin) or 160 μL (for RNase A) of additives in 10 mM Na-phosphate buffer (pH 7.0), and subsequently with 60 μL (for lysozyme, ChT, BSA, and subtilisin) or 120 μL (for RNase A) of PS particle solution.After incubation at 25°C for 1 h, the samples were centrifuged at 19000×g for 30 min. Protein concentration in the supernatant was then determined by A280 nm (A_280_) by spectrophotometry (V-630; Jasco Corp., Tokyo, Japan).

### Desorption of protein bound to polystyrene particles

A solution of 240 μL containing lysozyme was first mixed with 60 μL of PS particles in 10 mM Na-phosphate buffer (pH 7.0). After incubation for various periods, 150 μL of additives were added to the samples. After further incubation for 1 h, the samples were centrifuged at 19000×g for 30 min. Protein concentration in the supernatant was then measured. The final concentrations of lysozyme, PS particles, and additives were 0.067 mg/mL, 39 m^2^/L, and 500 mM, respectively.

## Results

**Figure 1:**
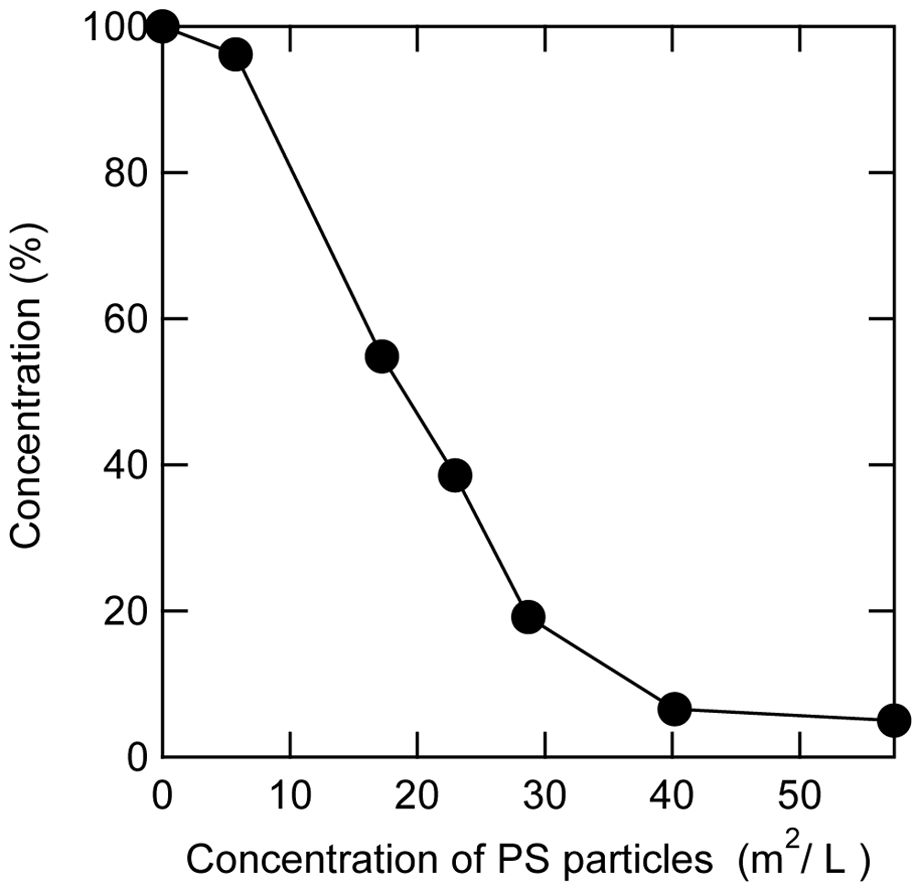
Adsorption of lysozyme to PS particles. The solutions containing 0–58 m^2^/L PS particles and 0.1 mg/mL lysozyme in 10 mM Na-phosphate buffer at pH 7.0 was incubated at 25°C for 1 h. After centrifugation, protein concentration in the supernatant was determined.

We first examined adsorption of lysozyme on polystyrene particles (PS particles) in the absence or presence of the following additives: Arg, Gdn, NaCl, Lys, Gly, and Glc. Lysozyme is a highly basic protein with a molecular mass of 14300 Da and an isoelectric point (p*I*) of 11.4. PS particles are composed of a polystyrene backbone and terminal sulfate groups. Thus, lysozyme and PS particles are oppositely charged at pH 7.0. To determine the appropriate ratio between lysozyme and PS particles, 0.1 mg/mL lysozyme in 10 mM Na-phosphate buffer (pH 7.0) was mixed with various concentrations of PS particles. The mixtures were centrifuged to remove protein-adsorbed PS particles after 1 h of incubation, and then the A_280_ of the supernatant was measured for determination of residual protein concentration. As shown in Figure 1, the lysozyme concentration in the supernatant decreased with increasing concentration of PS particles in a single exponential manner, an indication of equilibrium lysozyme adsorption. Almost all of the lysozyme was adsorbed above 40 m^2^/L with only ∼5% of protein remaining unbound. Therefore, we selected 58 m^2^/L PS particles for examination of the additive effects.

**Figure 2:**
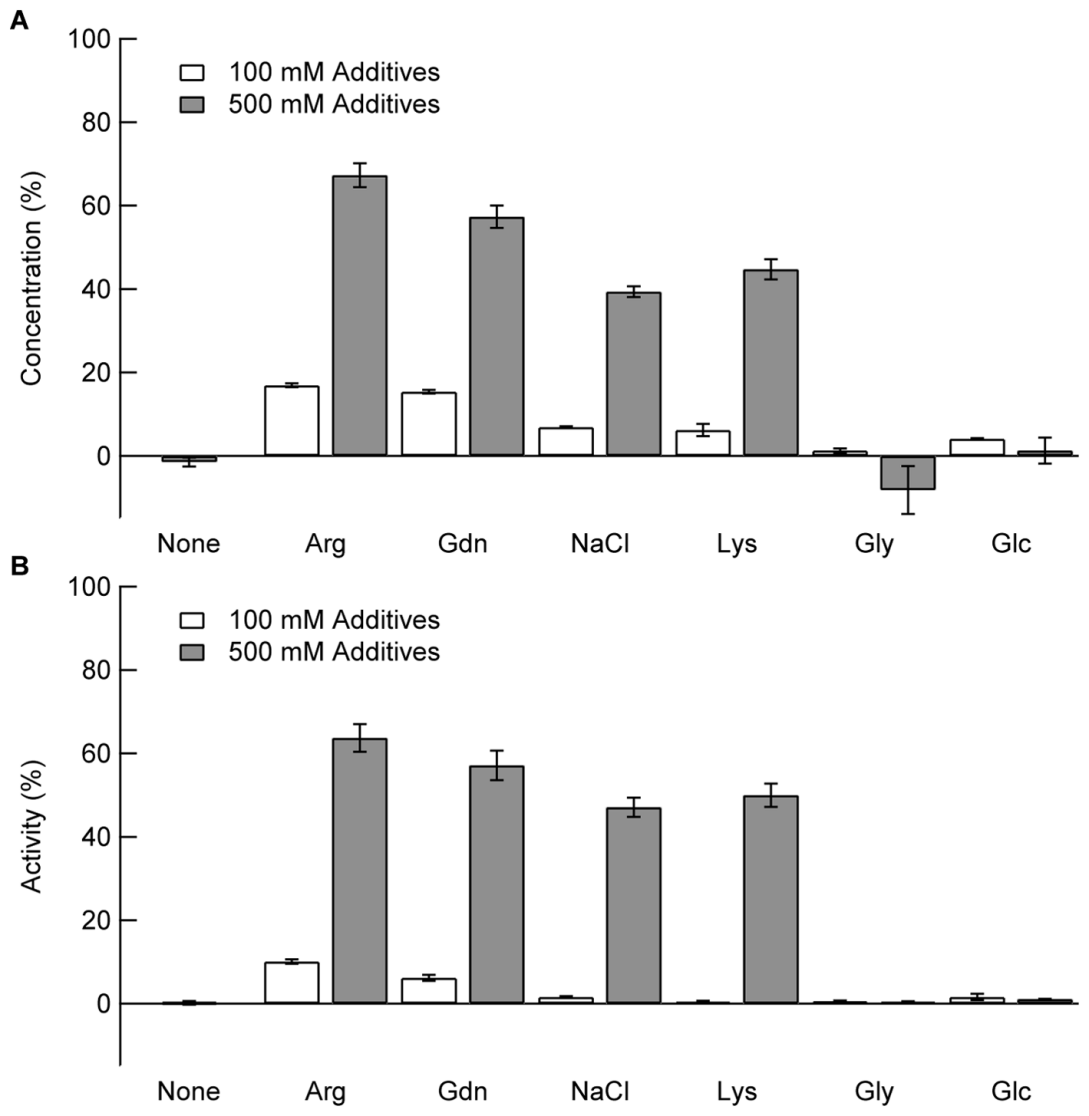
Effects of additives on lysozyme adsorption monitored by the concentration (A) and activity (B). The solutions containing lysozyme and additives were mixed with PS particles in 10 mM Na-phosphate buffer at pH 7.0, and then incubated at 25°C for 1 h. After centrifugation, protein concentration (A) and enzyme activity (B) in the supernatant were determined. The final concentrations of lysozyme and PS particles were 0.1 mg/mL and 58 m^2^/L, respectively.

Figure 2A shows the adsorption of lysozyme in the absence or presence of additives. Although the lysozyme concentration in the supernatant was zero in the absence of additives, the supernatant concentration increased to about 15% with the addition of 100 mM Arg and Gdn (Fig. 2A, white bars). At 500 mM, a much greater increase was observed in the presence of Arg and Gdn, but Arg was more effective. Arg at 500 mM prevented the adsorption of lysozyme to the PS particles by about 70%. The effects of NaCl and Lys in increasing supernatant protein concentration were second to those of Arg and Gdn. Gly and Glc were ineffective even at 500 mM.

Similarly, we examined the residual enzyme activity in the absence or presence of additives. As expected from the lack of lysozyme in the supernatant, there was no enzyme activity in the absence of the additives (see Fig. 2B, None). The dependence of enzyme activity on additives paralleled the dependence of lysozyme concentration (compare Fig. 2A and 2B). Enzyme activities were recovered with 100 mM Arg and Gdn, while no recovery was observed with addition of 100 mM NaCl, Lys, Gly, or Glc (Fig. 2B, white bars). Lysozyme showed the highest level of activity in the presence of 500 mM Arg, followed by 500 mM Gdn, Lys, and NaCl. Gly and Glc were ineffective at both 100 mM and 500 mM.

**Figure 3:**
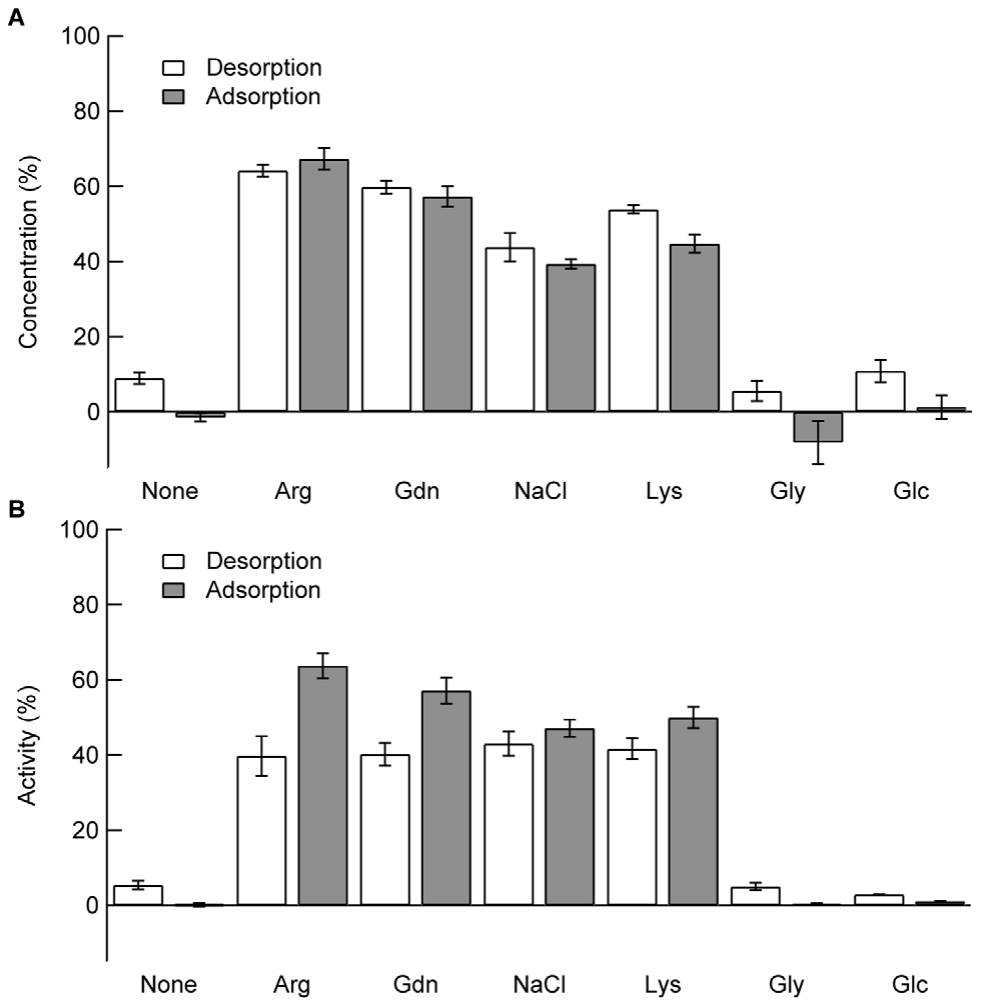
Effects of additives on lysozyme adsorption when the order of mixing was changed. The annotation “Desorption” (white bars) corresponds to the case where the additives were added after adsorption of lysozyme on the PS particles. The final concentrations of lysozyme, PS particles, and additives were 0.1 mg/mL, 58 m^2^/L, and 500 mM, respectively. The annotation “Adsorption” (gray bars) means that the additives were mixed with lysozyme before the addition of PS particles. The final concentrations of lysozyme, PS particles, and additives were 0.067 mg/mL, 38 m^2^/L, and 500 mM, respectively. After centrifugation of each sample, protein concentration (A) and enzyme activity (B) in the supernatant were determined.

In the above experiments, additives were present at the same time when lysozyme was mixed with the PS particles. That is, additives were first mixed with lysozyme solution, and subsequently with PS particles. In the next experiment, lysozyme and PS particles were first mixed, leading to complete binding of lysozyme. Then, additives were added to the above sample to desorb the bound lysozyme from the PS particles. As shown in Figure 3A and 3B, the addition of buffer solution slightly recovered both concentration and activity of lysozyme with ∼10% and ∼5%, respectively (compare first white bar with first gray bar). This recovery was likely due to the decreases in concentration of both lysozyme and PS particles; in this case, buffer solution was added to the solution containing 0.1 mg/mL lysozyme and 58 m^2^/L PS particles, resulting in 1.5-fold dilution. This dilution caused slight desorption of lysozyme bound to PS particles. A similar dependence on the order of mixing was seen for Gly and Glc. That is, when first equilibrated with lysozyme and PS particles, both protein concentration and enzyme activity slightly increased by Gly and Glc (Fig. 3A and 3B, white bars). For other additives, protein concentration showed marginal dependence on the order of mixing (Fig. 3A). The effectiveness of the additives on increase in protein concentration was Arg > Gdn > Lys > NaCl. The observations where the additives were added after adsorption clearly indicated that these additives, in particular Arg, can effectively desorb the bound lysozyme. On the other hand, the enzyme activity was lower than the increase in protein concentration when Arg, Gdn, Lys, and NaCl were added after adsorption (Fig. 3B). For example, when lysozyme was first bound to PS particles, protein concentration and enzyme activity in the supernatant were 60% and 40%, respectively, although both protein concentration and activity were identical (slightly under 60%) when lysozyme was first equilibrated with Arg. It is possible that pre-binding of lysozyme to PS particles caused irreversible denaturation, leading to inactivation. Therefore, Arg likely desorbed both active and inactive lysozyme, resulting in a greater increase in protein concentration.

**Figure 4:**
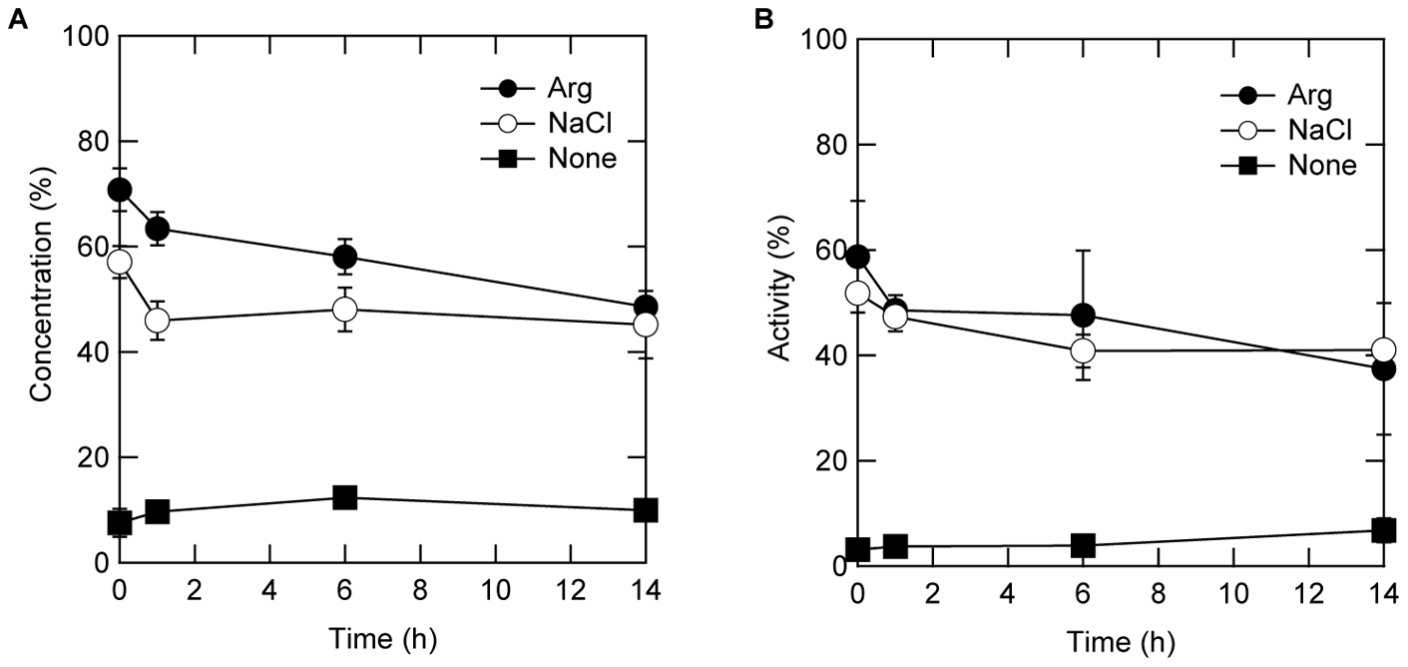
Time course of protein desorption (A) and enzyme activity (B) of lysozyme in the supernatant. The additives were added after adsorption of lysozyme on the PS particles. The final concentrations of lysozyme, PS particles, and additives were 0.067 mg/mL, 38 m^2^/L, and 500 mM, respectively.

The above results suggested that binding of lysozyme to the PS particles at least partially inactivated the protein. The mechanism of binding-induced inactivation was investigated by incubating lysozyme with PS particles in the absence of additives, which should result in complete binding. As shown in Figure 4, addition of buffer alone resulted in little increase in protein concentration or activity regardless of incubation time. Both Arg and NaCl desorbed lysozyme, but Arg was more effective immediately after binding. Both protein concentration and enzyme activity decreased in a similar time-dependent manner in the presence of additives, i.e., the effects of Arg and NaCl on both values were decreased by 20% and 10% after 14 h incubation, respectively.

**Figure 5:**
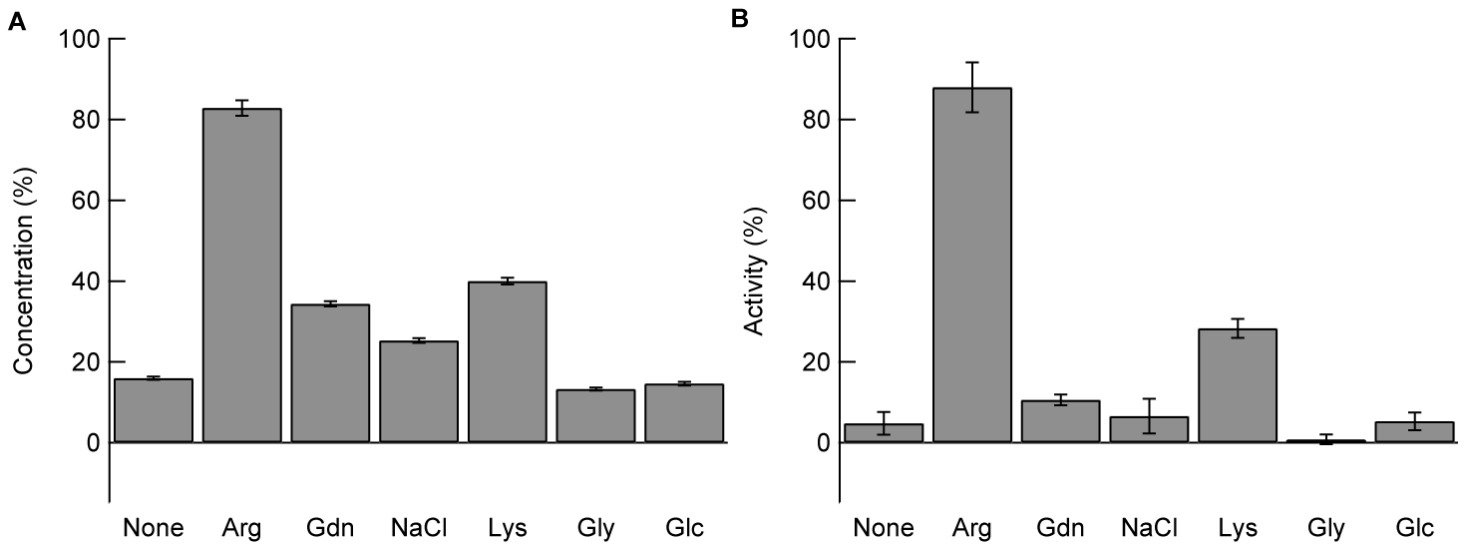
Effects of additives on ChT adsorption monitored by concentration (A) and activity (B). The solutions containing ChT and additives were mixed with PS particles in 10 mM Na-phosphate buffer at pH 7.0, and then incubated at 25°C for 1 h. After centrifugation, protein concentration (A) and enzyme activity (B) in the supernatant were determined. The final concentrations of ChT and PS particles were 0.1 mg/mL and 58 m^2^/L, respectively.

We examined ChT as another model protein with different structural properties from lysozyme to confirm the versatility of Arg as an adsorption suppressor. ChT has a molecular mass of 25000 Da and a p*I* of 8.8. Figure 5A shows the adsorption of ChT in the absence or presence of the additives. A small fraction of protein (∼15%) remained unbound in the supernatant in the absence of additives. The ChT concentration in the supernatant increased in the presence of 500 mM Arg, NaCl, Lys, and Gdn, similar to the results for lysozyme. In particular, Arg was extremely effective in preventing ChT binding to PS particles over 80%. Gly and Glc were ineffective as observed for lysozyme. The order of effectiveness was Arg > Lys > Gdn > NaCl > None > Gly > Glc. The enzyme activity in the supernatant was lower than the protein concentration with the exception of samples with addition of Arg (Fig. 5B), suggesting that Arg is effective in preventing not only protein binding but also activity loss in the case of ChT.

**Figure 6:**
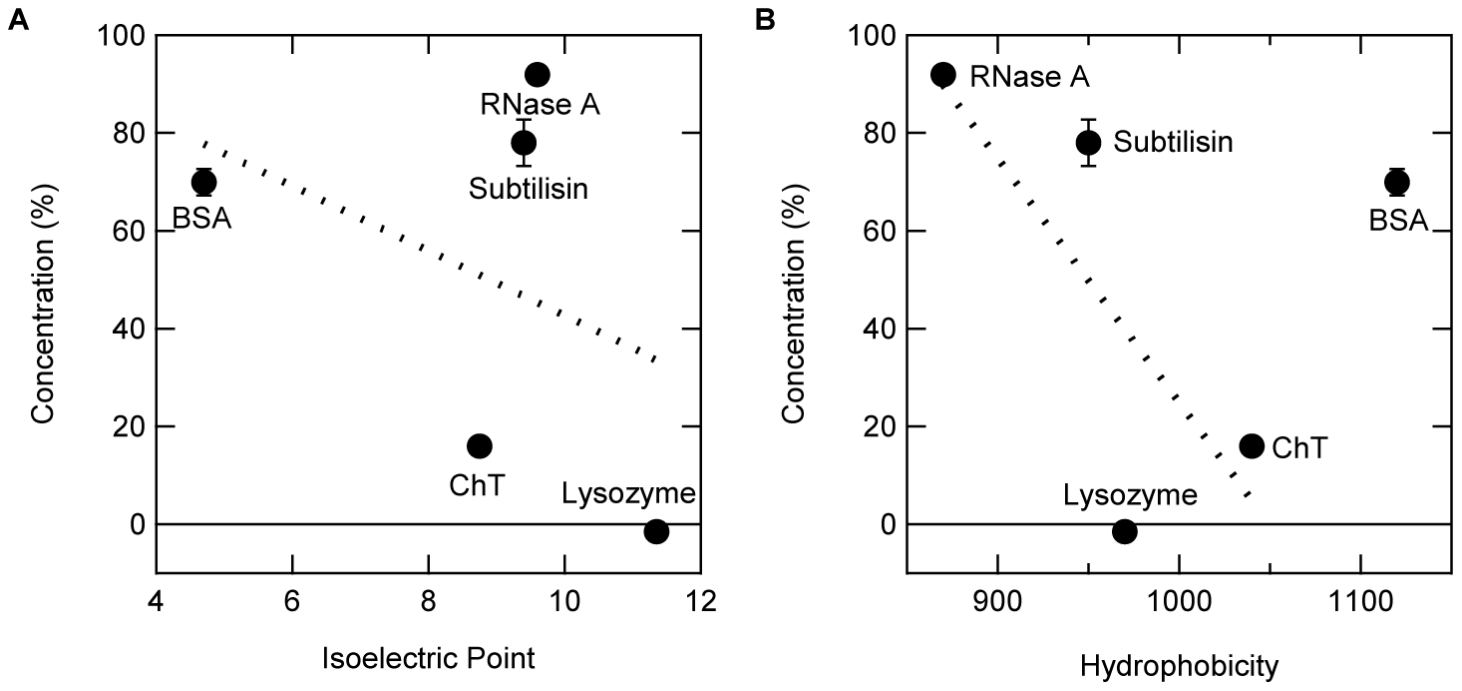
Relationships between protein adsorption and isoelectric point (A) or hydrophobicity (B) of proteins. The solutions containing 0.1 mg/mL proteins and 58 m^2^/L PS particles in 10 mM Na-phosphate buffer at pH 7.0 was incubated at 25°C for 1 h. After centrifugation, protein concentrations in the supernatant were determined.

PS particles are negatively charged at pH 7.0 and hence should bind the basic proteins to a greater extent. Figure 6 shows the relationship between protein adsorption and p*I* (A) or hydrophobicity (B) obtained using the same concentrations of proteins and PS particles. For this purpose, three more proteins, i.e., BSA (p*I*  = 4.7), subtilisin (p*I*  = 9.4), and RNase A (p*I*  = 9.6), were included. There appeared to be a poor correlation with p*I*. Among the basic proteins, subtilisin and RNase A did not bind as much as expected from their p*I*, perhaps due to their weak hydrophobicity. In addition, ∼30% of BSA was adsorbed, although there should be strong electrostatic repulsion between negatively charged BSA and PS particles. These observations indicated that their binding must overcome this unfavorable free-energy barrier most likely by hydrophobic interaction. Thus, the protein concentration was replotted against hydrophobicity of the protein [38]. As shown in Figure 6B, there appeared to be a negative correlation with hydrophobicity between basic proteins, meaning that more protein was bound with increasing hydrophobicity. Lysozyme deviated from the negative slope. This was expected, as it is extremely basic and hence charge–charge interaction overwhelms the hydrophobic contribution, leading to ∼100% binding. Acidic BSA deviated from this relation due to strong charge repulsion, which presumably offsets hydrophobic adsorption to the PS particles. Taken together, these data indicated that the amount of protein adsorption on PS particles depends on the balance of electrostatic and hydrophobic interactions.

**Figure 7:**
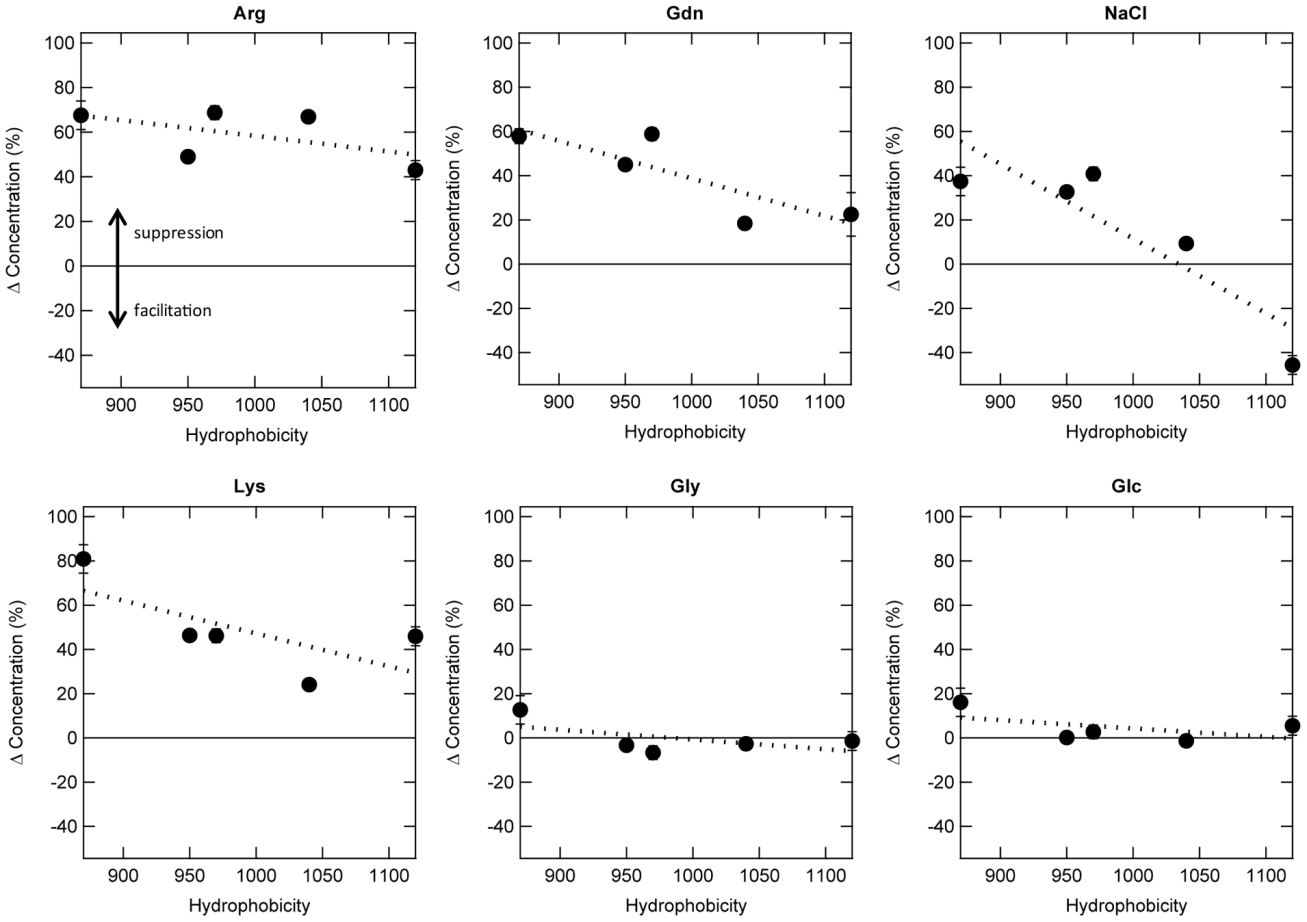
Effects of additives on protein adsorption as a function of protein hydrophobicity. The solutions containing proteins and 500 mM additives were mixed with PS particles in 10 mM Na-phosphate buffer at pH 7.0, and then incubated at 25°C for 1 h. After centrifugation, protein concentration in the supernatant was determined. The concentration of proteins and PS particles were as follows: (i) Lysozyme, ChT, and BSA–0.1 mg/mL protein and 58 m^2^/L PS particles; (ii) RNase A–0.1 mg/mL protein and 116 m^2^/L PS particles; (iii) Subtilisin–0.25 mg/mL protein and 58 m^2^/L PS particles.

The dependence between the effects of additives and protein hydrophobicity was evaluated by plotting the relative increase in recovery over the protein concentration observed in the absence of the additives against hydrophobicity (Fig. 7). Arg showed the greatest increase among the additives, and the suppressive effects were almost maintained when the hydrophobicity of proteins increased, i.e., it prevented protein adsorption well for both highly polar RNase A and hydrophobic BSA. Both Lys and Gdn followed Arg, but the effect decreased with increasing protein hydrophobicity as compared with Arg. NaCl prevented adsorption to some extent for polar proteins, but had no effect or even rather facilitated adsorption for hydrophobic proteins. Gly and Glc were ineffective.

## Discussion

Nonspecific protein adsorption to various surfaces causes not only loss of the protein but also potentially irreversible denaturation and enzyme inactivation. Such surfaces include polystyrene, glass test tubes, and chromatographic supporting materials. In this study, polystyrene particles (PS particles) were used as a model surface. The polymers that make up the PS particles are composed of a hydrophobic ethylene backbone, aromatic phenyl group, and terminal sulfate group, all of which can contribute to protein binding. The terminal sulfate groups occur due to polymerization reaction mechanism of polystyrene, although polystyrene is considered to be uncharged. Such terminal charges are exposed on the PS particles format and critical for effective dispersion of the PS particles. In other words, PS particles will aggregate and precipitate without the terminal charges that should be solvent exposed on the PS particles surface. Hydrophobic interaction through the backbone and aromatic side chain of PS particles played a critical role in protein adsorption on PS particles, as shown by the negative correlation with hydrophobicity between basic proteins toward protein adsorption, and binding of acidic BSA that has the same charge as PS particles by ∼30% (Fig. 6B). Terminal sulfate groups of PS particles generate a constellation of negative charges at the particle surface, which are also responsible for protein adsorption on PS particles by electrostatic interaction. Figure 6A shows the important role of electrostatic binding, as indicated by complete binding of basic lysozyme that has the opposite charge to PS particles and reduced binding of acidic BSA. All ionic additives, such as NaCl, Gdn, Lys and Arg, are monovalent and hence have an identical ionic strength at neutral pH. Thus, they should be equally effective in suppressing electrostatic interactions, as described below.

Among the additives used in this study, Arg was the most effective in preventing protein adsorption on PS particles. The mechanism of action of Arg involves suppression of hydrophobic binding that may play a role in ChT and BSA binding to the PS particles. Arg can also exert an effect on electrostatic interaction, primarily involved in lysozyme binding, due to its ionic properties, although its ionic character is about 50% that of NaCl at identical molar concentration. Finally, the most important contribution of Arg could be due to its strong interaction with aromatic structures. Protein bound through the aromatic side chains of PS particles can be effectively desorbed by competitive interaction of Arg with the aromatic structures of protein. At 1 M concentration range, protein hydration starts to play an important role in determining the overall interaction of additives with proteins and hence their effects on protein properties, here surface adsorption. Such overall interaction, termed preferential interaction, showed weak preferential exclusion of Arg from the protein surface, most likely due to steric exclusion of Arg [39]. The observed weak preferential exclusion of Arg is a reflection of protein hydration and its affinity for protein surface, as has been described above and yet no effect on protein stability [27], as supposed to protein stabilization by strongly excluded additives (e.g., sodium sulfate) and protein destabilization by preferentially bound additives (e.g., Gdn).

Both Gdn and Lys were also effective, although they showed weaker effects than Arg, as they are both ionic and can participate in aromatic-cation interactions similar to Arg [40]. Here, Arg was more effective than Gdn, as seen in many applications [27],[31]. While effective for certain proteins, NaCl was much less effective overall and even enhanced binding of such hydrophobic proteins as BSA. NaCl can suppress electrostatic interaction, but can enhance hydrophobic interaction due to its salting-out effectiveness: the observed preferential exclusion of NaCl was much stronger than that of Arg, implying no affinity of NaCl for protein surface [41]. As both Gly and Glc do not have ionic or hydrophobic characters and cannot participate in aromatic interactions, these additives were completely ineffective.

Additives not only inhibited adsorption but also caused desorption (Fig. 3A). Addition of additives after complete binding of lysozyme to PS particles desorbed the bound lysozyme from the PS particles. This desorption by Arg and NaCl occurred even 14 h after lysozyme binding to the PS particles and the protein concentration was fivefold higher than in the absence of additives. Moreover, the recovery of enzyme activity was sixfold higher than without additives after 14 h of binding. However, the value of the enzyme activity was lower when lysozyme was first bound to the PS particles in the absence of additives than when lysozyme was bound in the presence of additives. These observations indicated that a portion of lysozyme adsorbed on PS particles is deactivated. Moreover, protein concentration and enzyme activity decreased in a similar time-dependent manner when Arg and NaCl were mixed after lysozyme was bound to PS particles. These observations suggest that the amount of reversibly bound inactive proteins increased and further became irreversible. These results may be explained by assuming three populations of bound lysozyme, i.e., irreversibly bound inactive protein, reversibly bound inactive protein, and reversibly bound active protein. The latter can be fully recovered by Arg and NaCl. Arg can also recover some portion of irreversibly bound protein.

In summary, we have shown that the addition of Arg markedly suppresses protein adsorption on PS particles, which is a commonly-used material for vessels such as disposable test tubes and microtubes, and moreover can detach adsorbed proteins from PS particles. It has already been shown that Arg is one of the most versatile solution additives for proteins. Arg has a number of advantages in that it is a natural cell metabolite, biodegradable, nontoxic, and inexpensive. Arg has been used to enhance protein refolding, augment acid-induced viral inactivation, suppress protein aggregation during long-term storage, and enhance protein recovery during column chromatography. These effects of Arg may be at least in part due to its ability to suppress multiple interactions between proteins and chemical structures of solid surfaces as observed with the model PS particles.
